# Linear model for fast background subtraction in oligonucleotide microarrays

**DOI:** 10.1186/1748-7188-4-15

**Published:** 2009-11-16

**Authors:** K Myriam Kroll, Gerard T Barkema, Enrico Carlon

**Affiliations:** 1 Institute for Theoretical Physics, Katholieke Universiteit Leuven, Celestijnenlaan 200D, Leuven, Belgium; 2 Institute for Theoretical Physics, Utrecht University, Leuvenlaan 4, 3584 CE Utrecht, the Netherlands; 3 Instituut-Lorentz for Theoretical Physics, University of Leiden, Niels Bohrweg 2, 2333 CA Leiden, the Netherlands

## Abstract

**Background:**

One important preprocessing step in the analysis of microarray data is background subtraction. In high-density oligonucleotide arrays this is recognized as a crucial step for the global performance of the data analysis from raw intensities to expression values.

**Results:**

We propose here an algorithm for background estimation based on a model in which the cost function is quadratic in a set of fitting parameters such that minimization can be performed through linear algebra. The model incorporates two effects: 1) Correlated intensities between neighboring features in the chip and 2) sequence-dependent affinities for non-specific hybridization fitted by an extended nearest-neighbor model.

**Conclusion:**

The algorithm has been tested on 360 GeneChips from publicly available data of recent expression experiments. The algorithm is fast and accurate. Strong correlations between the fitted values for different experiments as well as between the free-energy parameters and their counterparts in aqueous solution indicate that the model captures a significant part of the underlying physical chemistry.

## Background

The analysis of microarray data has attracted continuous interest over the past years in the Bioinformatics community (see e.g. [1]). The problem consists in obtaining the gene expression level from the experimental measurements, which are the emitted fluorescence intensities from different sites in the array. On general grounds one expects that the experimental signal can be decomposed into two contributions:(1)

where *I*_SP_(*c*) is the specific signal due to the hybridization of the surface-bound probe sequence with a complementary target sequence. This quantity depends on the concentration *c *of the complementary strand in solution (target). The non-specific term *I*_bg _has different origins. It arises due to spurious effects such as incomplete hybridization where probe sequences bind to only partially complementary targets or due to other optical effects.

Models based upon the physical chemistry of hybridization (see e.g. [2]) predict a linear increase of the specific signal until saturation is approached. In case of highly expressed genes the specific part of the signal *I*_SP_(*c*) dominates the total signal intensity *I *and hence one can safely make the approximation *I *≈ *I*_SP_(*c*). For lowly expressed genes, as well as for sequences with a low binding affinity, the specific and the non-specific contribution to the total intensity can be of comparable magnitude. In this case an accurate estimate of *I*_bg _is crucial to draw reliable conclusions concerning the expression level; estimates based on the intensity distribution over the whole chip suggest that this is the case for roughly a quarter or half of the probes [3]. Once the background is calculated the gene expression level is then computed from background subtracted data *I *- *I*_bg_.

In this paper we present an algorithm for the calculation of the background level for Affymetrix expression arrays, also known as GeneChips. In these arrays the probe sequences come in pairs: for each perfect match (PM) probe, which is exactly complementary to the transcript sequence in solution, there is a second probe with a single non-complementary nucleotide with respect to the specific target. The latter is called mismatch (MM) probe.

Several algorithms for background analysis of Affymetrix chips are available. Some of these use the MM intensities as corrections for non-specific hybridization, while others rely on PM intensities only. For instance, the Affymetrix MAS 5.0 software (Microarray Analysis Suite version 5.0) uses the difference (*I*^PM ^- *I*^MM^) as estimator of the specific signal; an adjusted MM intensity (ideal MM) is used in the case the MM intensity exceeds the PM signal [4]. The Robust Multiarray Algorithm (RMA) [5] uses a different type of subtraction scheme which does not involve the MM intensities. The more recent version of this algorithm GCRMA performs background subtraction using information on the probe sequence composition through the calculation of binding affinities [6]. The position-dependent nearest-neighbor model (PDNN) [7] fits the background intensity using weight factors which depend on the position along the probe. The free energy parameters then enter in a nonlinear function. In the VSN algorithm [8] a generalized log transform is used to background correct the data. A study dedicated to the performance of different algorithms showed that the type of background subtraction used has a large effect on the global performance of the algorithms [9]. It is therefore not surprising that the background issue has attracted a lot of interest by the scientific community.

In this paper we present an algorithm for background estimation which combines information from the sequence composition and physical neighbors on the chip. This algorithm relies on previous work by the authors [10]. While the previous algorithm performed well with respect to the accuracy of the background estimation, the computational effort (per probe) involved was a severe limiting factor concerning its practical usability. The main cause of this significant computational effort was the iterative minimization of a cost function with nonlinear terms. The algorithm presented in this work involves a different cost function which is quadratic in the parameters. Its minimization can be performed via standard matrix computations of linear algebra. The algorithm is fast and accurate and is therefore suited for large scale analysis.

This paper is organized as follows. In Methods we discuss the optimization step from singular value decomposition and we provide the details of the selected cost function. In Results a test of the algorithm on about 360 Genechips from recent (2006 onwards) experiments from the Gene Expression Omnibus (GEO) is presented. Finally, the advantages of this scheme and its overall performance as background subtraction method is highlighted.

## Methods

### Approach

The general assumption is that the (natural) logarithm of the background intensity can be approximated by a function linear in some fitting parameters *ω*_*α*_. Once these parameters are set to their optimal values _*α *_an estimate of the background intensity for the *i*-th (*i *= 1, 2, ..., *N*_*dim*_) PM probe can be obtained as(2)

where *N*_*f *_is the number of fitting parameters. Ω_*iα *_is a sequence- and position-dependent element of the *N*_*dim *_× *N*_*f *_-dimensional matrix Ω, which will be defined below.

The optimized values for the model parameters _*α *_are obtained by minimizing the difference (of the logarithms) of the observed and estimated intensities(3)

i.e. solving a linear least square problem. The sum extends over the training set  which is a subset (with *K *elements) of the intensities of all annotated features. The choice of the elements of  will be discussed later (see Data Set - Parameter Optimization). The minimum is found by imposing vanishing partial derivatives of *S *w.r.t. *ω*_*α*_. This yields the following *N*_*f *_linear equations(4)

which can be rewritten as(5)

Here, we have introduced the matrix(6)

and the vector Γ_*α*_(7)

(Note that *M *= Ω^*T*^Ω is symmetric and dim(*M*) = *N*_*f *_× *N*_*f*_).

The solution of Eq. (5) is given by the vector(8)

of the optimal parameter values. If the matrix *M *is singular, *M*^-1 ^has to be replaced by its pseudoinverse *M*^+^which can be obtained by means of Singular value decomposition (SVD). In this work a standard SVD algorithm based on Golub and Reinsch is used (see e.g. [11,12]).

Due to the symmetry of *M *only half of the off-diagonal elements need to be generated, hence reducing the computational effort. For the chips tested in this paper with dimensions up to 1164 × 1164 features, the computational time on a standard PC (×86_64 Intel Core 2 Duo with 3 GHz, 3 GB RAM) required to estimate the background intensities is 8 to 10 seconds for the larger chips, and faster for the smaller ones.

This makes our algorithm an order of magnitude faster than our previous version [10], 3 to 5 times faster than GC-RMA, PDNN and MAS5, and about twice as slow as RMA and DFCM (Bioconductor packages were used for the testing). Note that for our algorithm, the time estimate includes both reading in the CEL-file and the background calculation, as it is done in one step.

This computation involves the generation of the matrix *M *and vector Γ (from Eqs. (6) and (7)), the SVD of *M *to solve Eq. (5) and the estimation of the background intensity for all PM probes through Eq. (2). Differently from other approaches in which the cost function is minimized by means of Monte Carlo methods [13] or other dynamical algorithms [10], the solution of SVD provides the exact minimum of the cost function Eq. (3). Hence, there is no risk in getting stuck in local minima different from the global one.

### Data Set - Parameter Optimization

As mentioned above, probes in Affymetrix form PM/MM pairs. Consider now a target sequence at a concentration *c *in solution. The analysis of Affymetrix spike-in data (see e.g. [14]) shows that not only the PM signal increases with increased target concentration *c *but also the MM intensity. This is an indication that a single MM nucleotide only partially prevents probe-target hybridization. Therefore the intensity of MM probes can also be decomposed in a non-specific and specific part as in Eq. (1). Supported by Affymetrix spike-in data analysis, our assumption is that the non-specific part of the hybridization is about equal for PM and MM probes: . The specific part of the signal is different in those two cases; equilibrium thermodynamics suggests a constant ratio , independent of the target concentration, as observed in experiments [3].

These insights are useful for the selection of probes for the optimization set  in Eq. (3):  includes all MM probes whose intensities are below a certain threshold *I*_0 _and whose corresponding PM intensities also fulfill  <*I*_0 _(a similar selection criterion was recently used by Chen et al. [15]). The threshold *I*_0 _is chosen on the basis of the total distribution of the intensities.  contains a significant fraction of the mismatch probes: typically 35%. Since the specific signal of MM intensities is lower than that of their corresponding PM's, they provide more reliable information on the background. The coordinates and sequences of the probes in  are then fitted to the intensities of these probes yielding the parameters *ω*. With those newly acquired parameters *ω *the background signal of all MM probes is estimated based upon the assumption .

### The matrix Ω

The choice of the matrix elements of Ω is dictated by input from physical chemistry as well as by the architecture of the microarray. Different schemes involving different choices for Ω with a varying number of parameters *N*_*f *_were tested. Given a choice of Ω and in particular the number *N*_*f *_of fitting parameters, the accuracy of the background estimation is reflected by the value of *S *from the minimization of Eq. (3). While the addition of fitting parameters always yields lower values of *S*, a too large set of fitting parameters runs the risk of "overfitting". The final choice of Ω is a compromise between a minimization of *S *and the use of the smallest possible set of parameters.

In the present model the number of parameters is *N*_*f *_= 50. Similarly to the previous work [10] these parameters can be split into two groups: a first group describes the correlation of the background intensities with features which are physical neighbors on the chip; the second group are nearest-neighbor parameters which describe affinities for non-specific hybridization to the chip.

#### Physical Neighbors on the Chip

The first 18 parameters *ω*_*α *_describe the correlation of the background intensity with physical neighboring sequences in the array. Let (*x*_*i*_, *y*_*i*_) be the coordinate of the *i*-th MM sequence *s*(*i*). Then, its eight neighbors are located at (*x*_*i *_± 1, *y*_*i*_), (*x*_*i *_± 1, *y*_*i *_± 1), and (*x*_*i*_, *y*_*i *_± 1). As there is evidence that base-specific interactions (purine/pyrimidine asymmetry) might influence the hybridization process in general [16], we furthermore distinguish between probes whose central nucleotide is either A/G (purine) or C/T (pyrimidine). Then, the corresponding matrix elements in case of purines can be written as(9)

with(11)

and

so that the intensities of the neighboring features explicitly enter the calculation of the background intensity of (*x*_*i*_, *y*_*i*_) as matrix elements Ω_*iα *_(2 ≤ *α *≤ 9). In analogy to Eqs. (9,10) we define Ω_*iα *_with 10 ≤ *α *≤ 18 corresponding to the sequences with a central pyrimidine.

#### Nearest-Neighbor Free Energy Parameters

The second contribution to the background model arises from the sequence composition. Let us first label the 16 dinucleotides according to the order {*CC*, *GC*, *AC*, *TC*, *CG*, *GG*, *AG*... *TT*}. We then define the next 16 matrix elements as(12)

where(13)

according to the order given above. The sum runs over all the 24 dinucleotides along a probe sequence.

The matrix element *Ω*_*αi*_ is equal to the number of dinucleotides of a given type in the sequence *s*(*i*). For instance, if the sequence *s*(*i*) contains 4 dinucleotides of type *CC *and 2 of type *GC*, then Ω_*i*,19 _= 4 and Ω_*i*,20 _= 2. Hybridization thermodynamics predicts log *I *∝ Δ*G *where Δ*G *is the hybridization free energy.

In the nearest-neighbor model [17] the free energy is written as a sum of dinucleotide terms. Therefore, the parameters *ω*_*α *_(19 ≤ *α *≤ 35) are the analogues of the free energy parameters of the nearest-neighbor model.

#### Position-Dependent Nearest-Neighbors

Finally, we consider the possibility that the hybridization strength is "modulated" along the sequence by a parabolic weight as(14)

where *l*_*m *_= 12.5, i.e. each dinucleotide is given a parabolic weight according to its position relative to the center at *l*_*m *_of the sequence. Thus, possible "unzipping" effects of the DNA-RNA duplex are approximately accounted for by Eq. (14).

The introduction of a position-dependence effect is in analogy with work done by other groups [7,16,18,19]. However, we do not introduce a position-dependent weight for each position along the 25-mer sequences. Instead, we limit ourselves to a parabolic modulation of the parameters along the chain, which drastically reduces the number of parameters involved in the model.

#### Invariances

Given the definition of Ω above it can be shown that Eq. (5) permits multiple solutions. Therefore, the optimal parameters _*α *_are not unique. Consider a set of optimal parameters _*α *_which minimizes the cost function *S *(Eq. (3)). Let us then add a constant *λ *to the 16 nearest-neighbor parameters(15)

From Eq. (12) we obtain(16)

because Ω_*iα *_counts the frequency of the dinucleotide *α *in the sequence corresponding to feature *i *and because there are 24 dinucleotides in a 25-mer probe sequence. Now, also consider(17)

while  = _*α *_for all other *α*. We conclude that the reparametrization of Eqs. (15) and (17) yields(18)

since the shifting of Eq. (17) compensates the one introduced by Eq. (16). This reparametrization, valid for any real *λ*, leaves *S *invariant, and produces a zero eigenvalue of the matrix *M *of Eq. (6).

Similarly, one can verify that there is at least a second zero eigenvalue: a shift of the position-dependent nearest-neighbor parameters  = _*α *_+ *λ *(for 35 ≤ *α *≤ 50) as well as of ,  leaves *S *invariant. To obtain the latter equations Eq. (14) and  have to be applied.

Having zero eigenvalues, the matrix *M *is therefore not invertible; the SVD thus provides the appropriate pseudo-inverse as discussed above. Accidental degeneracies or quasi-degeneracies of M could also occur, yielding eigenvalues close to zero in machine precision. These are, however, rare, and were actually never found in the calculations presented here.

## Results

We analyzed a total of 366 CEL-files which are publicly available from the GEO server http://www.ncbi.nlm.nih.gov/geo. Table 1 gives an overview of the distribution of CEL-files over the twelve different organisms considered in this study. The array size for an organism might vary depending on the GSE accession number, since the most recent Affymetrix chips tend to use smaller features, thus more probes can be accommodated on the same surface area. For instance the Human HGU-133A contains 712^2 ^features, while the Human Genome U133 Plus 2.0 Array goes to 1164^2 ^features. The last column of Table 1 lists the attained minimum value _*min*_, which ranges from 0.017 for Escheria Coli to 0.11 for Oryza Sativa. _*min *_estimates the mean squared deviation of the logarithm of the estimated background intensity from the actual background value. For instance _*min *_= 0.01 corresponds to a 10% deviation, while _*min *_= 0.1 corresponds to a 37% deviation. Table 2 provides a summary of the optimal parameters as calculated from the Singular Value Decomposition for the minimization of the cost function of Eq. (3). The parameters _1 _and _10 _are associated to constant intensities for purines and pyrimidines. Their magnitudes are not unique due to the reparametrization as discussed in Eqs. (15) to (18). If information on neighboring probes is disregarded, the value of *S *typically increases by 45%; if the sequence information is not directly used, then it will increase by 52%. In the following we analyze the parameters associated to the local physical neighbors and to the nearest-neighbor free energy.

**Table 1:** Overview over organisms and number of CEL-files analyzed

Organism	GEO #	Chiptype (dimension)	# files	_*min*_
A. Thaliana	GSE4847	ATH1-121501 (712 × 712)	18	0.0259
	GSE7642	ATH1-121501 (712 × 712)	12	0.0544
	GSE9311	ATH1-121501 (712 × 712)	8	0.0546

C. Elegans	GSE6547	Celegans (712 × 712)	25	0.0361
	GSE8159	Celegans (712 × 712)	7	0.0396

D. Melanogaster	GSE3990	Drosophila_2 (732 × 732)	6	0.0620
	GSE6558	DrosGenome1 (640 × 640)	24	0.0605

D. Rerio	GSE4859	Zebrafish (712 × 712)	8	0.0357

E. Coli	GSE11779	E_coli_2 (478 × 478)	3	0.0869
	GSE2928	Ecoli (544 × 544)	12	0.0172
	GSE6195	E_coli_2 (478 × 478)	4	0.0664

H. Sapiens	GSE10433	HG-U133A_2 (732 × 732)	12	0.0757
	GSE5054	HG-U133A (712 × 712)	20	0.0392
		HG-U133A_2 (732 × 732)		

M. Musculus	GSE7148	HG-U133A (712 × 712)	14	0.0296
	GSE8514	HG-U133_Plus_2 (1164 × 1164)	15	0.0738
	GSE11897	MOE430A (712 × 712)	11	0.0640
		MOE430B (712 × 712)		
		Mouse430_2 (1002 × 1002)		
	GSE6210	Mouse430_2	12	0.0594
	GSE6297	Mouse430_2	24	0.0325

O. Sativa	GSE15071	Rice (1164 × 1164)	20	0.1157

R. Norvegicus	GSE4494	RG_U34A (534 × 534)	59	0.0488
	GSE7493	Rat230_2 (834 × 834)	9	0.0497
	GSE8238	Rat230_2 (834 × 834)	4	0.0640

S. Aureus	GSE7944	S_aureus (602 × 602)	6	0.0746

S. Cerevisiae	GSE6073	YG_S98 (534 × 534)	12	0.0283
	GSE8379	YG_S98 (534 × 534)	8	0.0180

X. Laevis	GSE3368	Xenopus_laevis (712 × 712)	20	0.0514

**Table 2:** Optimized parameter values as obtained from the minimization of Eq. (3).

	A. Thaliana			C. Elegans		D. Melanogaster		D. Rerio
GEO no	GSE4847	GSE7642	GSE9311	GSE6547	GSE8159	GSE3990	GSE6558	GSE4859
_1_	0.178	0.196	0.261	0.379	0.520	0.270	0.421	0.338
_2_	0.050	0.056	0.042	0.047	0.050	0.022	0.025	0.041
_3_	0.051	0.056	0.041	0.034	0.042	0.024	0.024	0.050
_4_	-0.013	-0.012	-0.013	-0.012	-0.016	-0.005	-0.011	-0.012
_5_	-0.013	-0.011	-0.012	-0.010	-0.012	-0.003	-0.010	-0.008
_6_	0.186	0.198	0.224	0.168	0.271	0.228	0.195	0.140
_7_	0.004	0.005	0.003	0.003	0.005	0.001	0.005	0.006
_8_	0.002	0.009	0.003	0.002	0.005	0.001	0.006	0.002
_9_	0.013	0.027	0.010	0.008	0.008	0.009	0.012	0.014
_10_	-0.174	-0.192	-0.258	-0.375	-0.517	-0.267	-0.418	-0.334
_11_	0.063	0.060	0.059	0.058	0.062	0.042	0.057	0.068
_12_	0.069	0.064	0.064	0.052	0.059	0.046	0.061	0.075
_13_	-0.017	-0.015	-0.016	-0.011	-0.012	-0.012	-0.013	-0.016
_14_	-0.018	-0.016	-0.019	-0.010	-0.009	-0.011	-0.014	-0.012
_15_	0.258	0.299	0.328	0.301	0.459	0.336	0.316	0.246
_16_	0.004	0.006	0.005	0.005	0.009	0.002	0.008	0.007
_17_	0.002	0.007	0.003	0.003	0.006	0.001	0.007	0.004
_18_	0.013	0.025	0.012	0.012	0.015	0.009	0.016	0.016

_19_	0.068	0.138	0.143	0.149	0.155	0.159	0.217	0.140
_20_	0.052	0.191	0.169	0.090	0.157	0.124	0.152	0.140
_21_	-0.021	-0.038	0.006	-0.033	0.030	-0.060	-0.033	-0.069
_22_	-0.021	0.051	0.056	-0.025	0.072	-0.084	0.003	-0.068
_23_	-0.032	-0.162	-0.147	-0.038	-0.121	-0.060	-0.094	-0.104
_24_	0.041	0.075	0.072	0.024	0.015	0.030	0.048	0.035
_25_	-0.063	-0.221	-0.162	-0.122	-0.140	-0.172	-0.200	-0.190
_26_	-0.060	-0.130	-0.119	-0.118	-0.109	-0.173	-0.154	-0.181
_27_	0.000	-0.008	-0.047	0.035	-0.041	0.052	0.035	0.055
_28_	0.036	0.148	0.076	0.062	0.063	0.105	0.096	0.128
_29_	-0.026	-0.058	-0.069	-0.046	-0.044	-0.065	-0.085	-0.052
_30_	-0.050	-0.032	-0.093	-0.065	-0.052	-0.106	-0.102	-0.078
_31_	0.057	0.037	0.056	0.082	0.012	0.133	0.103	0.130
_32_	0.093	0.180	0.173	0.125	0.112	0.211	0.181	0.221
_33_	0.011	-0.074	-0.021	-0.008	-0.026	0.015	-0.036	0.001
_34_	-0.009	-0.017	-0.016	-0.023	-0.014	-0.035	-0.047	-0.022
Corr. Coeff	0.775	0.686	0.738	0.814	0.700	0.754	0.826	0.705

_35_	0.020	0.016	0.016	0.019	0.013	0.016	0.012	0.018
_36_	0.020	0.010	0.012	0.021	0.011	0.016	0.016	0.017
_37_	0.025	0.025	0.022	0.029	0.019	0.028	0.027	0.031
_38_	0.024	0.019	0.019	0.028	0.016	0.028	0.023	0.031
_39_	0.026	0.036	0.034	0.030	0.030	0.027	0.031	0.033
_40_	0.021	0.021	0.020	0.026	0.021	0.022	0.023	0.026
_41_	0.028	0.039	0.035	0.036	0.031	0.035	0.038	0.039
_42_	0.027	0.032	0.031	0.035	0.028	0.033	0.033	0.038
_43_	0.023	0.024	0.026	0.024	0.023	0.019	0.021	0.021
_44_	0.020	0.013	0.017	0.022	0.015	0.015	0.017	0.016
_45_	0.024	0.028	0.028	0.030	0.024	0.027	0.029	0.029
_46_	0.025	0.025	0.029	0.030	0.024	0.028	0.029	0.030
_47_	0.020	0.022	0.020	0.022	0.021	0.015	0.019	0.016
_48_	0.017	0.012	0.012	0.019	0.013	0.010	0.014	0.011
_49_	0.023	0.029	0.026	0.028	0.023	0.023	0.028	0.026
_50_	0.023	0.023	0.023	0.028	0.021	0.024	0.026	0.026
Corr coeff	-0.679	-0.618	-0.679	-0.759 18	-0.631	-0.683	-0.783	-0.658

Magnitudes and signs of the parameters are approximately constant across different experiments. The correlation coefficients are the Pearson correlations between the nearest-neighbor parameters and the free energy parameters measured in hybridization experiments in aqueous solution [20].

### Parameters of physical neighbors in the Chip

The parameters _2 _to _9 _and _11 _to _18 _describe the coupling of the background intensities to the physically neighboring features on the chip. As already mentioned, our estimate of the PM background is based on the non-specific intensity of the MM sequence. An Affymetrix chip is designed such that MM and PM are found in rows at equal *y*-coordinates. In addition, given a PM at (*x*, *y*), the corresponding MM feature is at (*x*, *y *+ 1).

**Figure 1:**
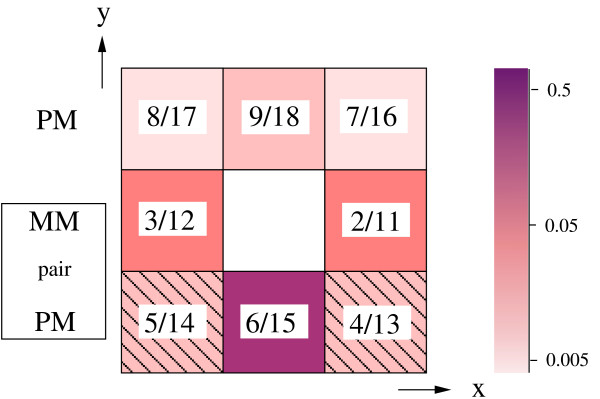
**Correlations with neighboring features**. Schematic representation of the neighboring parameters on the array. The inset numbers indicate the corresponding parameter(s) _*α*_, e.g. 3/12 represents _3_, _12 _respectively. _2 _through _9 _are related to purines, _11 _to _18 _to pyrimidines. The bar to the right gives the intensity scale of the parameters. The dashed pattern indicates negative values. Box on the left indicates the rows of corresponding PM and MM pairs. The central feature (position (*x*, *y*)) is a MM; its corresponding PM is located just below it (*x*, *y *- 1), its intensity has the strongest correlation with the central MM feature. The features in (*x *± 1, *y *- 1)

Figure 1 schematically represents the influence of the neighboring intensities on the background value. The strength of the correlation of the eight neighboring spots (i.e. the magnitude and sign of the corresponding _*α*_) with the central MM feature is indicated by the color. The numbers in the figure identify the associated parameters _*α*_. For example _6 _and _15 _are associated to the intensity at (*x*, *y *- 1) with respect to the reference MM intensity with coordinates (*x*, *y*). There does not seem to be any evidence that the middle-nucleotide classification in purines and pyrimidines reveals any insight concerning the background. Instead, our results show that the absolute values of two "corresponding" (purine-pyrimidine pair) _*α *_'s are generally of the same order of magnitude. Across all species, we find typical average outputs of

Since _6_, _15 _respectively, reflects the correlation of the MM signal with its corresponding PM, as expected its magnitude is greatest among all parameter values. Next, the MM intensity shows strong correlations with its direct nearest neighbors positioned at (*x *± 1, *y*), i.e. _2/11 _and _3/12_. Hence, the stronger the MM-neighboring intensities, the stronger their influence on the background signal of MM. However, in order to somehow compensate for strong MM-neighboring signals which might be caused by the presence of complementary target sequences, parameters _4/13 _and _5/14 _have negative sign. The remaining three parameters, i.e. the top three neighbors limit their influence to a basically negligible minimum which appears to indicate the weak sequence correlation in *y*-direction as previously found. The results indicate that the influence of neighboring intensities on the background noise is significant. In fact, our analysis shows that ≈ 30% of log  are constituted by neighboring probes in terms of absolute intensities (see Table 3). It appears that for a few probes, the latter might even play a crucial role. It is unlikely that the correlations summarized in Fig 1 could be explained only as simple optical effects, as light from bright probes spilling into their neighborhood. Indeed, optical effects should produce an isotropic correlation pattern in the *x *and *y *directions which is not seen in our analysis. Another cause for this correlation might be that neighboring probes share common sequences, as is the case in Affymetrix chips [10].

**Table 3:** Influence of neighboring spot on background intensity in % of eight (randomly chosen) CEL-files of different organisms.

X. Laevis	74500.CEL 35%	76190.CEL 37%
C. Celegans	201989.CEL 43%	201994.CEL 52%

H. Sapiens	263931.CEL 41%	263930.CEL 39%

S. Cerevisiae	207569.CEL 29%	207570.CEL 29%

### Nearest-Neighbor Parameters

The parameters _*α *_(19 ≤ *α *≤ 34) are the analogues of the nearest-neighbor free energy parameters. The nearest-neighbor model is commonly used to study the thermodynamics of hybridization of nucleic acids in solution (see e.g. [17]). In this model it is assumed that the stability and thus the hybridization free energy Δ*G *of a dinucleotide depends on the orientation and identity of the neighboring base pairs. For RNA/DNA duplexes there are 16 hybridization free energy parameters which were measured in aqueous solution by Sugimoto et al. [20].

Recent experiments [21] focusing on specific hybridization show a good degree of correlation between the hybridization free energies in solution and those directly determined from microarray data. Concerning background data, we also expect a certain degree of correlation between the parameters _*α *_(19 ≤ *α *≤ 34) and their corresponding Sugimoto free energy parameters.

**Figure 2:**
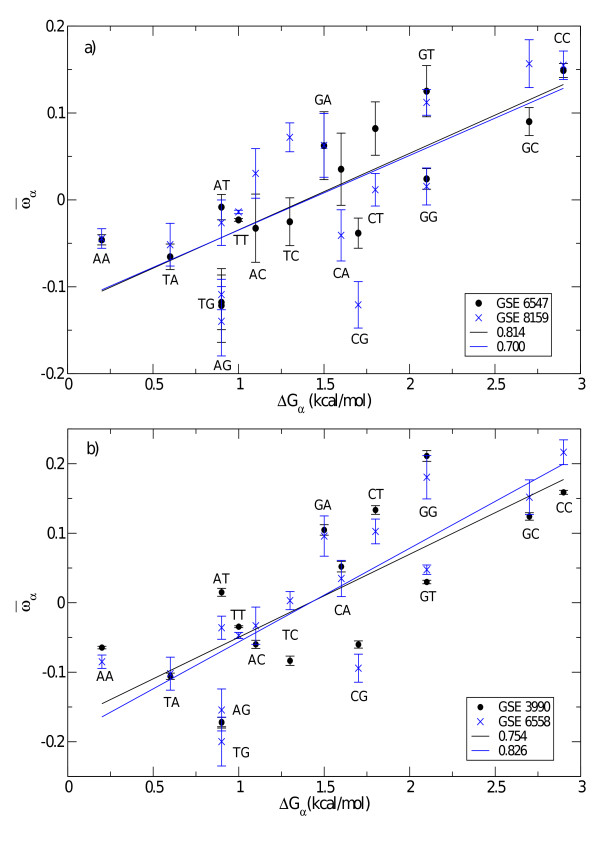
**Fitted vs. solution hybridization free energies**. Pair Parameters _*α *_(19 ≤ *α *≤ 34), as obtained from the minimization of Eq. (4) as function of Δ*G*_*α*_, the nearest-neighbor stacking free energy obtained from DNA/RNA hybridization in solution [20]. Plots refer to two GSE training data sets of a) C. Elegans and b) Drosophila Melanogaster. Each point on the *y*-axis is the averaged value over all CEL-files representing its GSE-set. The error bars are the standard deviation. Notation of DNA pairs are from 5' to 3' end. The straight lines are linear fits; correlation coefficients of each GSE set in legend.

In order to test the relationship between the experimentally determined so-called Sugimoto parameters and our results, we calculate the correlation coefficient between these two sets. The results are reported in Table 2. In general, the correlation coefficients vary between 0.53 and 0.83 with a median value of 0.71. Figure 2 shows two typical results for C. Elegans and D. Melanogaster. Both plots indicate that the relationship between _*α *_(19 ≤ *α *≤ 34) and the nearest-neighbor free energy parameters of [20] is approximately linear.

### Position-dependent nearest neighbor parameters

**Figure 3:**
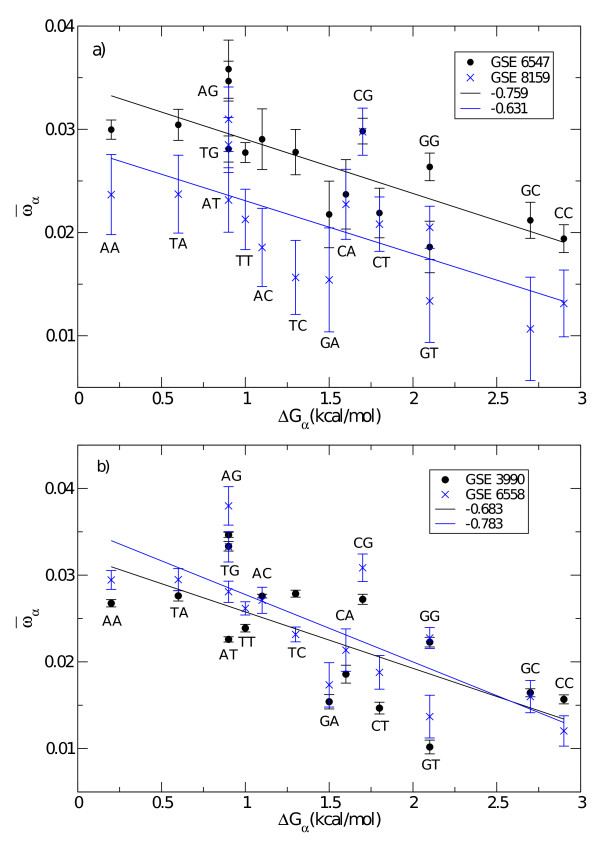
**Fitted vs. solution hybridization (position dependent) free energies**. Same as in Fig. 2 for parabolic pair parameters, i.e. Δ*G*_*α *_vs. _*α *_(35 ≤ *α *≤ 50) for a) C. Elegans and b) D. Melanogaster.

Figure 3 shows a plot of position-dependent nearest-neighbor parameters _35 _to _50 _as obtained from the minimization of the cost function of Eq. (3) for four different sets of experiments. The data are plotted as function of the corresponding nearest neighbor free energy parameters of [20]. We note that in all experiments shown there is a negative correlation between the two data sets. The parameters _35 _to _50 _reflect the difference in effective free energy between the ends and the middle of the probe sequence. Since weakly binding probes suffer more from end-effects (unzipping, etc.), _35 _to _50 _and their corresponding nearest-neighbor free energy parameters are negatively correlated. Hence, the negative correlation in Fig. 3 and those observed in all other cases (see correlation coefficients reported in Table 2) indicates that the dominant contribution to the background intensity comes from the middle nucleotides. This conclusion complies with other types of analysis which use position-dependent free energy parameters (see e.g. [7,16]).

### Comparing estimated vs. measured background

**Figure 4:**
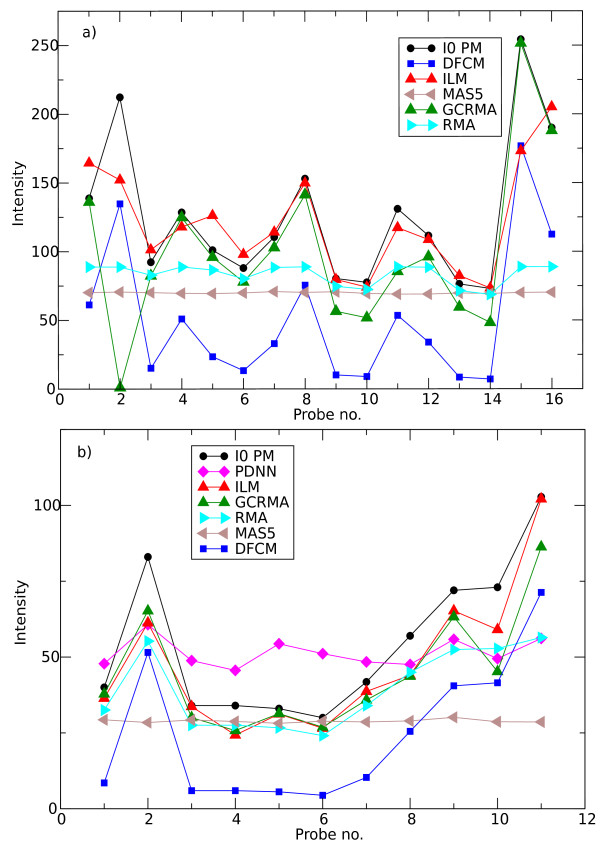
**Computed vs. true background (spike-in experiments)**. Experimental results (*I*0*P M*) and theoretical predictions of the algorithms discussed here for a) probeset 37777_at (HGU95A, 1521a99 hpp_av06.CEL) and b) 209606_at (HGU133A,12_13_02_U133A_Mer_Latin_Square_Expt10_R1.CEL) spiked-in at concentration *c *= 0, i.e. absent from the hybridizing solution. All comparison were performed using freely available packages from the Bioconductor project.

In this section we compare the estimated background signal (as given in Eq. (2)) with the intensities of given probe sets corresponding to non-expressed genes in the samples analyzed. We start with publicly available data taken from spike-in experiments on HGU95A chips http://www.affymetrix.com where genes have been spiked-in at known concentrations, ranging from 0 to 1024 pM (picoMolar). The data at 0 pM correspond to the absence of transcript in solution. Figure 4a compares the measured and predicted background for probeset 37777_at. Except for probes 2 and 15 for which the measured signal is higher than the predicted background, there is a nice agreement between our prediction and the experimental background intensity: the standard deviation of the absolute difference between the intensity of the PM and *I*^est ^is 28 in Affymetrix intensity units. Also shown are the background estimations as obtained with other algorithms (the data for PDNN is missing in Fig. 4a due to the unavailability of one of the supporting files from Bioconductor packages). Figure 4b presents the same information for probeset 209606_at. Here, the standard deviation is 7 in Affymetrix intensity units.

**Figure 5:**
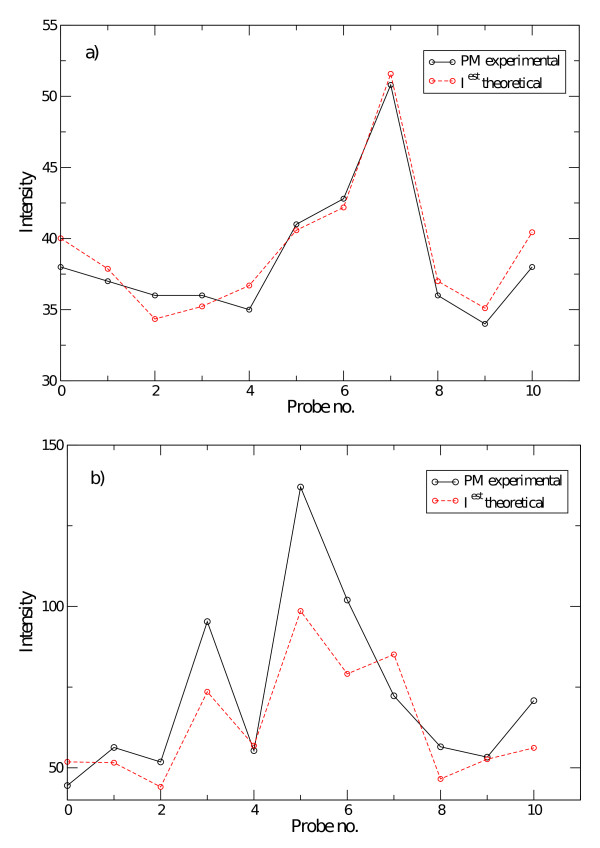
**Computed background vs. low expressed genes data**. Experimental results (solid black line) and theoretical prediction (dashed red line) of probeset a) 256610_at (A. Thaliana, GSE4847, GSM109107.CEL) and b) 175270_at (C. Elegans, GSE8159, GSM201995.CEL).

Next, we go beyond the spike-in experiments. To select non-expressed genes we considered probe sets with very low expression values as obtained from the RMA algorithm. In Figure 5 the PM intensities as well as the calculated background signal of two probesets from A. Thaliana and C. Elegans experiments are shown. The absolute intensities of both probesets are very low. As a consequence, we can safely assume these genes are not expressed and hence any measured signal can be attributed to the background. Figures 4 and 5 both show that the present model captures the essentials of and correctly predicts background intensities. Both Figures are representative for the CEL-files analyzed in this work.

## Discussion

We have presented a background subtraction scheme for Affymetrix GeneExpression arrays which is both, accurate and usable on a standard ×86_64 Intel Core 2 PC. The algorithm centers around a cost function which is quadratic in its fitting parameters. This allows for a rapid minimization, through linear algebra, in particular through singular value decomposition. The accuracy of the present algorithm is very similar to that of a background algorithm previously presented by the authors [10]. The latter had been tested on Affymetrix spike-in data and its performance was compared to background schemes such as MAS5, RMA and GCRMA. Regarding spike-in data, the analysis had shown that the proposed algorithm is definitely more accurate than background computations done with MAS5 and RMA, but also improves on GCRMA [10].

The proposed algorithm has two categories of fitting parameters. The first category exploits correlations between features which are neighbors on the chip. The second category is based on the strong similarity between probe-target hybridization and duplex stability in solution, and involves stacking free energies in analogy to those in the nearest-neighbor model. Existing algorithms are either of the first [4] or the second [6,7,9] category, but not both.

The background subtraction scheme has been tested on 360 GeneChips from publicly available data of recent expression experiments. Since the fitted values for the same parameters in different experiments do not show much variation, the algorithm is robust and can be easily transferred to other experiments. Due to its speed and accuracy the present method is suited for large scale computations. An R-package integrating the background analysis scheme with the computation of expression values from background subtracted data will be made freely available to the community (a preliminary version of this package can be found in http://itf.fys.kuleuven.ac.be/~enrico/ilm.html). The performance of this approach is discussed in [22].

## Competing interests

The authors declare that they have no competing interests.

## Authors' contributions

EC and GB planned and supervised the research. KMK wrote the code for data analysis and analyzed the data. KMK and EC wrote the paper.
